# Epidemiological study of antimicrobial-resistant bacteria in healthy free-ranging bantengs (*Bos javanicus*) and domestic cattle

**DOI:** 10.14202/vetworld.2023.1796-1802

**Published:** 2023-09-13

**Authors:** Adithep Konputtar, Montira Yossapol, Tarathip Phaechaiyaphum, Phaphatsorn Manyuen, Nannapas Suetrong, Nuntita Ruksachat, Tarid Purisotayo

**Affiliations:** 1 Faculty of Veterinary Sciences, Mahasarakham University, Maha Sarakham, Thailand; 2 Veterinary Infectious Disease Research Unit, Mahasarakham University, Maha Sarakham, Thailand; 3 Department of National Parks, Wildlife and Plant Conservation, Bangkok, Thailand

**Keywords:** antimicrobial resistance, banteng, cattle, extended-spectrum beta-lactamase, human-wildlife interface, wildlife

## Abstract

**Background and Aim:**

Antimicrobial-resistant microorganisms (ARMs) have been increasing among wild animals. Interactions occurring at the interface between wildlife, humans, and livestock can lead to the transmission of ARMs. Thus, the prevalence of ARMs in wild and domestic animals should be determined to address and prevent this issue. This study aimed to determine the resistance patterns of cefotaxime (CTX)-resistant *Escherichia coli* and identify the presence of extended-spectrum beta-lactamase (ESBL) genes in ESBL-producing *E. coli* among a population of wild banteng (*Bos javanicus*) and domestic cattle kept on farms located close to the Lam Pao non-hunting area, Kalasin province, Thailand.

**Materials and Methods:**

Forty-five fecal samples were taken from wild bantengs inhabiting the Lam Pao non-hunting area in Thailand, alongside 15 samples from domestic cattle. Bacterial culture, triple sugar iron, and motile indole lysine tests were conducted to identify *E. coli*. A polymerase chain reaction (PCR) was conducted for specific confirmation. MacConkey agar supplemented with 2 μg/mL of CTX was used to identify CTX-resistant *E. coli*, which would be used to identify ESBL production based on a double-disk synergy test. Extended-spectrum beta-lactamase-producing samples were subjected to disk diffusion tests to determine resistant patterns, and the sizes of PCR bands and DNA sequencing were used to differentiate ESBL gene types.

**Results:**

All samples tested positive for *E. coli*. Forty-five isolates from 15 banteng samples and three isolates from one domestic cattle sample displayed CTX-resistant and ESBL-producing traits. The banteng and domestic cattle populations exhibited nine and three distinct resistant patterns, respectively. The PCR results indicated that the banteng isolates harbored the following genes: *Cefotaxime-M1* (n = 38), *CTX-M9* (n = 5), and the *SHV* group (n = 2). All three isolates from the domestic cattle sample contained the *CTX-M1* gene. Classification of ESBL genes based on the DNA sequences of the banteng isolates showed the characteristics of *CTX-M15* (n = 20), *CTX-M55* (n = 6), *CTX-M14* (n = 5), and *CTX-M79* (n = 1). The three domestic cattle isolates exhibited the characteristics of *CTX-M15*, *CTX-M55*, and *CTX-M79*.

**Conclusion:**

Despite no previous antibiotic applications, approximately one-third of the banteng samples displayed CTX resistance, indicating ARM contamination within the ecosystem. The similarity in ESBL genes between the banteng and domestic cattle populations suggests potential gene transmissions between these animal groups. However, the initial source of ARMs remains unclear, as the banteng population exhibited more ESBL genes than the domestic cattle, suggesting the possibility of multiple ARM sources. These findings raise concerns because the banteng population inhabits an area that is an important source of freshwater and nourishes the entire north-east region of Thailand and other South-east Asian countries, including Laos, Cambodia, and Southern Vietnam.

## Introduction

The global human population has been rapidly increasing [1], necessitating the expansion of land uses for anthropogenic activities, such as agriculture to provide sufficient food resources. This expansion has caused habitat loss for wild animals, creating new wildlife-human-livestock (WHL) interfaces [2]. These interfaces have raised various public health concerns [3, 4], including the potential transmission of antimicrobial-resistant microorganisms (ARMs) between humans, livestock, and wildlife [3]. The emergence of ARMs in wild species poses several challenges and warrants the identification (ID) of potential sources and the mechanisms behind such transmissions. Inappropriate drug use has significantly contributed to the emergence of ARMs [3], particularly in non-human applications (e.g., livestock and companion animals). This is because antibiotics are heavily used as precautionary measures for high-risk animals and as growth promoters for healthy livestock, often mixed with their feed [5]. These applications illustrate violations of rational drug use proposed by the World Health Organization to prevent ARM emergence [6]. Thus, ARMs are likely to emerge in livestock and potentially spread to wildlife and ecosystems [7].

The emergence of ARMs has complicated the treatment of infectious diseases, often leading to treatment delays and/or failures. Beta-lactam antibiotics have been extensively prescribed in livestock due to their broad-spectrum activities [8]. These antibiotics primarily disrupt cell wall synthesis, resulting in bacterial death [9]. However, ARMs can produce beta-lactamase enzymes that hydrolyze the beta-lactam ring of these antibiotics [9], essentially degrading their pharmacological effects. The emergence of ARMs is primarily due to genetic mutation and selection [10]; however, genetic materials that encode antimicrobial-resistant genes (ARGs) can also be acquired from other microorganisms through horizontal gene transfer (HGT) [11]. The HGT mechanism is crucial because environments contaminated with ARM and non-pathogenic bacteria can serve as ARG reservoirs, facilitating the indirect transmission of these genes, even without the coexistence of the organisms at the WHL interfaces. *Escherichia coli* is a common flora that inhabits the gastrointestinal tracts of healthy organisms, with some strains found to exhibit pathogenicity and toxigenesis [12]. The previous study by Rupp and Fey [13] have shown that *E. coli* plasmid harbors genes encoding ampicillin-hydrolyzing beta-lactamases (e.g., TEM-1, TEM-2, and SHV-1) and cephalosporins-hydrolyzing extended-spectrum beta-lactamases (ESBLs). In this study, we focused on ESBL-producing *E. coli* strains crucial for the development of drug resistance [13]. Extended-spectrum beta-lactamase genes confer resistance to third-generation cephalosporins, which have been extensively used in veterinary practices for their broad-spectrum effects [8]. With over 150 documented ESBLs, they are differentiated from other ampicillin-hydrolyzing beta-lactamases by their ability to hydrolyze oximino-cephalosporins [14]. Most *E. coli* strains can tolerate extreme environmental changes and survive adverse conditions for months [15]. Thus, non-pathogenic *E. coli* strains are excellent reservoirs of ESBLs before these genes are transmitted to susceptible species.

This study aimed to identify the types of ESBLs present in ESBL-producing *E. coli* strains isolated from a free-ranging population of bantengs (*Bos javanicus*) in the Lam Pao non-hunting area (Kalasin, Thailand) and domestic cattle in neighboring human communities. The banteng, listed as “endangered” on the red list of threatened species of the International Union for Conservation of Nature since 2014, is estimated to have only 4000–8000 individuals left in their natural habitats [16]. Identifying ESBL profiles in wild bantengs and domestic animals would address the spillover of ARMs between wild and domestic animals, facilitating further studies aimed at preventing this issue at other WHL interfaces.

## Materials and Methods

### Ethical approval

This study was approved by the Institutional Animal Care and Use Committee, Mahasarakham University (IACUC-MSU), Thailand (Approval number IACUC-MSU-32/2021). Works involving legally protected animals and areas were approved by the Department of Natural Parks, Wildlife, and Plant Conservation, Thailand (MNRE 0907.4/439).

### Study period and location

The study was conducted from August 2021 to October 2022. The Lam Pao non-hunting area was established in 1988 with an area of approximately 338 km^2^ in Kalasin Province, North-east Thailand. Of the total area, three square kilometers have been inhabited by a population of about 100 free-ranging bantengs as well as other wild species such as Siamese hares (*Lepus peguensis*), various species of squirrels, Asian palm civets (*Paradoxurus hermaphroditus*), and migratory and non-migratory bird species [17]. This area is known for the local communities as Suan Sa-On (GPS coordinates 16.61345, 103.45910). The north, south, and west boundaries of Suan Sa-On are surrounded by the Pao River, and the east is adjacent to the land and human communities.

### Sample collection

**Figure-1:**
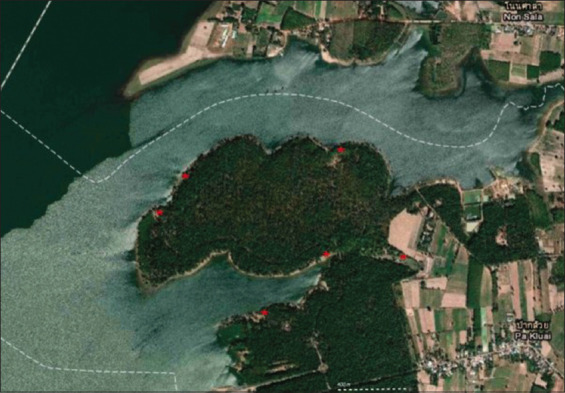
Map of Suan Sa-On non-hunting area (Courtesy of Geo-Informatics Center for Thailand). The six sampling sites of bantengs samples are shown as red asterisks. Note that the area is surrounded by local agricultural communities and the Pao River.

Fecal samples from the bantengs were collected from six locations (Figure-1) within the non-hunting area in August 2021. The locations were selected based on the preferred grazing areas of the bantengs, which were identified using observational records. Fecal samples from domestic cattle were collected from nearby farms within a 3-km radius of the non-hunting area that had records of antibiotic use. To avoid contamination, approximately 20 g of fresh feces from the middle portions that did not come into contact with the environment (i.e., air and soils) were sampled. Each sample was packaged individually in a sealed plastic bag and labeled with a unique sample ID number (Supplementary data). Overall, 45 banteng and 15 domestic cattle samples were collected. All samples were then preserved in ice-cooled containers (~4°C) and promptly transferred to the microbiology laboratory at the Faculty of Veterinary Sciences, Mahasarakham University, where they were refrigerated until further processing.

### Identification of *E. coli*

*Escherichia coli* strains were identified based on colony characteristics (i.e., pink, flat, smooth, and round) and biochemical tests. Colonies that showed characteristics consistent with *E. coli* on MacConkey agar (Himedia^®^, Mumbai, India) were considered potential colonies and verified using triple sugar iron (TSI) [18] and motile indole lysine (MIL) tests [19]. Three colonies (hereafter referred to as isolates) were selected from each sample that tested positive for both TSI and MIL, and these isolates were cultured on nutrient agar (Himedia^®^) and incubated at 37°C for 24 h. An isolate was collected using an inoculated loop and mixed with 400 μL of distilled water in a microcentrifuge tube. Next, the tube containing the isolate was boiled in 100°C water for 10 min and left to cool at room temperature (28–30°C). Centrifugation was conducted at 18,000× *g* and 4°C for 10 min. The supernatant (approximately 300 μL for each sample) was collected and kept at −20°C as a DNA template for polymerase chain reaction (PCR) analysis.

The PCR was performed for each sample using 6.25 μL of OnePCR^®^ Ultra solution (GeneDirex, USA), 0.05 μL of the ECO-1 primer, 0.05 μL of the ECO-2 primer (Table-1), 1.0 μL of DNA templates, and 5.65 nuclease-free water. The PCR protocol followed these steps: (1) Initial denaturation at 94°C for 1 min, (2) denaturation at 98°C for 3 s, (3) annealing at 50°C for 30 s, (4) an extension at 74°C for 35 s, (5) steps 2–4 repeated for 35 cycles, (6) a final extension at 74°C for 2 min, and (7) elongation at 45°C for 2 s. Polymerase chain reaction products were visualized using 1.5% agar rose gel electrophoresis in a 1× Tris-acetate-EDTA (TAE) buffer, compared to a 100 base pairs (bp) DNA ladder (VC 100 bp DNA ladder, Vivantis, Malaysia) and positive controls. Samples displaying a band of 585 bp were considered positive.

**Table-1:** Primers used for the identification ESBL genes.

Genes	Primers	Sequences (5’ ⟶ 3’)	Amplicon sizes (bp)
*Escherichia coli malB*	ECO-1	GACCTCGGTTTAGTTCACAGA	585
	ECO-2	CACACGCTGACGCTGACCA	
*bla*TEM	MultiTSO-T_for	CATTTCCGTGTCGCCCTTATTC	800
	MultiTSO-T_rev	CGTTCATCCATAGTTGCCTGAC	
*bla*SHV	MultiTSO-S_for	AGCCGCTTGAGCAAATTAAAC	713
	MultiTSO-S_rev	ATCCCGCAGATAAATCACCAC	
*bla*OXA	MultiTSO-O_for	GGCACCAGATTCAACTTTCAAG	564
	MultiTSO-O_rev	GACCCCAAGTTTCCTGTAAGTG	
*bla*CTX-M-1	CTXmGr1_F	AAA AAT CAC TgC gCC AgT TC	415
	CTXmGr1_R	AgC TTA TTC ATC gCC ACg TT	
*bla*CTX-M-2	CTXmGr2_F	CgA CgC TAC CCC TgC TAT T	552
	CTXmGr2_R	CCA gCg TCA gAT TTT TCA gg	
*bla*CTX-M-9	CTXmGr9_F	CAA AgA gAg TgC AAC ggA Tg	205
	CTXmGr9_R	ATT ggA AAg CgT TCA TCA CC	
*bla*CTX-M-8/25	CTXmGr8/25_for	AAC RCR CAG ACG CTC TAC	326
	CTXmGr8/25_rev	TCG AGC CGG AAS GTGTYT T	
Sequencing primers	SeqCTX-M-1-for	GACTATTCATGTTGTTGTTATTTC	923
	SeqCTX-M-1-rev	TTACAAACCGTTGGTGACG	
Sequencing primers	SeqCTX-M-9-for	ATGGTGACAAAGAGAGTGCAACGG	876
	SeqCTX-M-9-rev	TCACAGCCCTTCGGCGATGATTCT	

ESBL=Extended-spectrum beta-lactamase, CTX=Cefotaxime

### Bacterial culture and ID of cefotaxime (CTX)-resistant *E. coli*

To identify CTX-resistant *E. coli*, each sample was cultured on a Petri dish containing MacConkey agar (Himedia) supplemented with 2 μg/mL of CTX. The samples were then incubated at 37°C for 24 h. Only the *E. coli-*like colonies were selected for further confirmation, while the rest were preserved in tryptone soya broth with 10% glycerol at −20°C. The TSI [18] and MIL [19] tests were conducted, and samples positive for both tests were considered CTX-resistant *E. coli*.

### Identification of ESBL-producing CTX-resistant *E. coli* using the double-disk synergy test

The samples categorized as CTX-resistant *E. coli* were cultured in individual Petri dishes containing nutrient agar (Himedia). Three growth colonies were selected from each sample (three isolates per sample) and individually diluted in 0.85% NaCl to achieve a turbidity equivalent to 0.5 McFarland standard. A sterile cotton swab was immersed in the solution and then spread on Mueller-Hinton agar (MHA; Himedia). On an MHA Petri dish, an amoxicillin/clavulanate (20/10 μg/disk, respectively) disk (Oxoid, UK) and a CTX (30 μg/disk) disk (Oxoid) were placed at the center with a distance of 2.5 cm between them. Incubation was conducted at 37°C for 16–24 h, and the increase in the inhibition zone between both disks was considered an indicator of ESBL production [20].

### Disk diffusion test to determine resistant patterns

A drug susceptibility test was conducted for samples confirmed to be ESBL-producing. Each sample was cultured on nutrient agar (Himedia) and incubated at 37°C for 24 h. A colony was selected and diluted in 0.85% NaCl to achieve a turbidity equivalent to 0.5 McFarland standard. A sterile cotton swab was immersed in the solution and spread on a dish containing MHA (Himedia). Disks containing the following antibiotics were used for the drug susceptibility test: meropenem (MER) (10 μg), gentamicin (GEN; 10 μg), tetracycline (TET; 30 μg), ciprofloxacin (CIP; 5 μg), nalidixic acid (NAL; 30 μg), chloramphenicol (CHL; 30 μg), and sulfamethoxazole/trimethoprim (SXT; 23.75/1.25 μg). These antibiotics were selected from drugs commonly used in the area. To determine resistant patterns, the results were interpreted following the zone diameter interpretive criteria recommended by the Clinical and Laboratory Standards Institute [21].

### Identification of ESBL gene groups using the PCR technique

DNA extraction was conducted on the isolates identified as ESBL-producing and CTX-resistant, which served as DNA templates for subsequent PCR procedures. Extended-spectrum beta-lactamase genes were identified using the multiplex PCR technique. To identify *CTX-M* genes for each isolate, a PCR reaction mixture of 13 μL was prepared, which contained the following: 6.25 μL of 2X ViRed Taq Mastermix^®^ (Vivantis); 0.05 μL of the CTXmGr1_F, CTXGr1_R, CTXmGr2_F, CTXmGr2_R, CTXmGr9_F, CTXmGr9_R, CTXmGr8_F, CTXmGr25_F, and CTXmGr8/25_R primers (Table-1); 1.0 μL of DNA templates; and 5.30 μL of nuclease-free water. The PCR (Proflex^®^, Applied Biosystem, Bangkok) protocol followed these steps: (1) Initial denaturation at 94°C for 2 min, (2) denaturation at 98°C for 10 s, (3) annealing at 55°C for 30 s, (4) an extension at 72°C for 30 s, (5) steps 2–4 repeated for 25 cycles, and (6) a final extension at 72°C for 7 min. The PCR products were visualized using 1.5% of agar rose gel electrophoresis in a 1× TAE buffer. Extended-spectrum beta-lactamase gene groups were identified based on the sizes of PCR products relative to a 100 bp DNA ladder (VC 100 bp DNA ladder^®^, Vivantis) and positive controls of different groups.

To identify the TEM, SHV, and OXA gene groups for each isolate, a PCR solution of 10 μL was prepared. This solution contained 10 μL of 2X ViRed Taq Mastermix (Vivantis); 0.05 of the MultiTSO-T_for, MultiTSO-T_rev, MultiTSO-S_for, MultiTSO-S_rev, MultiTSO-O-for, and MultiTSO-O_rev primers (Table-1); 1.0 μL of DNA templates; and 3.7 μL of nuclease-free water. The same protocol used to conduct the PCR reactions and determine amplicon sizes was followed to identify the *CTX-M* genes.

### Identification of ESBL genes using DNA sequencing

To identify ESBL genes, sample preparation for DNA sequencing was conducted for each isolate using 10 μL of 2X ViRed Taq Mastermix (Vivantis); 0.05 μL of the SeqCTX-M-1_for, SeqCTX-M-1_rev, SeqCTX-M-9_for, and SeqCTX-M-9_rev primers (Table-1); 2.0 μL of DNA templates; and 7.90 μL of nuclease-free water. The same amplification protocol employed to identify ESBL gene groups using the PCR technique was followed. For quality control, 2.0 μL of PCR products were subjected to 2% agar rose gel electrophoresis (i.e., validation of *E. coli* based on amplicon sizes and confirmation of DNA materials). The PCR product from each isolate that showed a well-defined electrophoretic band with an appropriate size was selected to represent each resistant pattern. These PCR products were submitted for DNA sequencing (Solgent Co. Ltd., South Korea) and were examined for ESBL genes using the basic local alignment search tool [22] available on the National Center for Biotechnology Information website (https://blast.ncbi.nlm.nih.gov/Blast.cgi).

## Results

### Identification of *E. coli*

Based on the TSI and MIL tests, as well as the PCR analyses, all 135 isolates from the 45 banteng samples and 45 isolates from the 15 domestic cattle samples were confirmed as *E. coli* (Supplementary data).

### Bacterial culture and ID of CTX-resistant and ESBL-producing *E. coli*

Among the 45 banteng samples, 15 were identified as CTX-resistant *E. coli*, accounting for 33.3% of the total banteng samples (Table-2). These samples were labeled with the following IDs: BJ10, BJ12, BJ13, BJ17, BJ18, BJ23, BJ24, BJ26, BJ28, BJ31, BJ33, BJ34, BJ35, BJ42, and BJ44 (Supplementary data). Conversely, only one (ID: DC01) out of the 15 domestic cattle samples was CTX-resistant, representing 6.7% (Supplementary data).

**Table-2:** Percentages of samples that were deemed as CTX-resistant *Escherichia coli*.

Animals	Number of samples	Percentage of CTX resistance	Percentage of ESBL-producing samples
Bantengs	45	33.3 (15/45)	33.3 (15/45)
Domestic cattle	15	6.7 (1/15)	6.7 (1/15)

ESBL=Extended-spectrum beta-lactamase, CTX=Cefotaxime

The double-disk synergy test confirmed ESBL production in all 15 CTX-resistant banteng samples. Similarly, the only CTX-resistant sample among the domestic cattle was confirmed to be ESBL-producing. These results accounted for 33.3% and 6.7% of the total banteng and domestic cattle samples, respectively (Table-2).

### Disk diffusion test to determine resistant patterns

The 15 banteng samples deemed CTX-resistant and ESBL-producing exhibited the following resistant patterns (Table-3): CTX-TET-SXT-NAL-CHL-GEN (ID: BJ10), CTX-TET-SXT-NAL-CHL-CIP (IDs: BJ23 and BJ26), CTX-TET-SXT-NAL-CHL (ID: BJ28), CTX-TET-CHL-GEN (ID: BJ13), CTX-TET-CHL (ID: BJ31), CTX-TET-MER (ID: BJ35), CTX-SXT-GEN (ID: BJ33), CTX-TET (IDs: BJ24, BJ42, and BJ44), and CTX (IDs: BJ12, BJ17, BJ18, and BJ34). However, the three isolates from the CTX-resistant and ESBL-producing domestic cattle samples showed different resistant patterns (Table-3): CTX-TET-NAL-CHL (DC013), CTX-SXT-MER-NAL (DC012), and CTX-TET (DC011).

**Table-3:** Resistant patterns revealed from the disk diffusion test of the CTX-resistant ESBL-producing *Escherichia coli*.

Resistant pattern	Sample IDs
CTX-TET-SXT-NAL-CHL-GEN	BJ10
CTX-TET-SXT-NAL-CHL-CIP	BJ23, BJ26
CTX-TET-SXT-NAL-CHL	BJ28
CTX-TET-CHL-GEN	BJ13
CTX-TET-CHL	BJ31
CTX-TET-MER	BJ35
CTX-SXT-GEN	BJ33
CTX-TET	BJ24, BJ42, BJ44, DC011
CTX	BJ12, BJ17, BJ18, BJ34
CTX-TET-NAL-CHL	DC013
CTX-SXT-MER-NAL	DC012

BJ=Banteng samples, DC=Domestic cattle samples, ESBL=Extended-spectrum beta-lactamase, CTX=Cefotaxime, GEN=Gentamicin, TET=Tetracycline, CIP=Ciprofloxacin, NAL=Nalidixic acid, CHL=Chloramphenicol, SXT=Sulfamethoxazole/trimethoprim, MER=Meropenem

### Identification of ESBL gene groups using the PCR technique

Forty-five banteng isolates were obtained from the 15 CTX-resistant and ESBL-producing samples. Among these isolates, 84.5% (38) belonged to the *CTX-M1* group, while 11.1% (5) and 4.4% (2) were categorized under the *CTX-M9* and *SHV* groups. All three isolates (100%) obtained from one domestic cattle sample were classified under the *CTX-M1* group (Supplementary data).

### Identification of ESBL genes using DNA sequencing

Thirty-two banteng isolates and three domestic cattle isolates were selected for sequencing based on their well-defined electrophoretic bands with appropriate sizes. *Cefotaxime-M15* was detected in 20 banteng isolates and one domestic cattle isolate, while *CTX-M55* was observed in six banteng isolates and one domestic cattle isolate. Furthermore, *CTX-M14* was observed in five banteng isolates, and *CTX-M79* was observed in one banteng and one domestic cattle isolate (Supplementary data).

## Discussion

To the best of our knowledge, this study is the first to identify ESBL-producing *E. coli* among the wild banteng population of Thailand. This population has never been treated with antibiotics, suggesting the potential sharing of ARMs between bantengs and domestic cattle. The existence of these organisms in other wild species has been reported worldwide [7, 23, 24]. The first publication discussing the spread of cephalosporin-resistant bacteria in wild animals (e.g., wild boars, *Sus scrofa*) was published in 2004 [25]. Subsequently, the number of related studies steadily increased, with a notable acceleration between 2016 and 2020, during which approximately 60% of these studies were published [23]. The *CTX-M1* group identified in this study exhibited the highest prevalence among free-ranging bantengs, consistent with previous studies by Homeier-Bachmann *et al*. [24], Torres *et al*. [26], Formenti *et al*. [27], Sasaki *et al*. [28] and Niumsup *et al*. [29] on other wild ungulates and healthy individuals in rural Thailand regions. The prevalence of *CTX-M1* and *CTX-M9* observed in this study is consistent with the findings reported in other studies involving domesticated animals [30, 31] and humans [28, 29] in South-east Asia. In addition, most ESBL genes identified through DNA sequencing were shared between the bantengs and domestic cattle. Only one gene (i.e., *CTX-M14*) observed in the bantengs was absent in domestic cattle. This consistency across studies and the shared genes between these animal groups highlight the potential for ARG transmission among humans, wildlife, and domestic animals.

However, regarding the number of gene counts, the banteng samples exhibited more ESBL genes than the domestic cattle samples. Homeier-Bachmann *et al*. [24] reported a contrary finding, with fewer ESBL genes observed in wild animals than in domestic species. The dissimilarity in our study is likely due to the differing sample sizes between the wild banteng (45 samples) and domestic cattle (15 samples) populations, as a smaller sample size limits the detection of less common ESBLs. However, regardless of sample size, our results suggest that the bantengs did not solely acquire ESBL genes from the domestic cattle. Instead, they suggest the existence of other ESBL sources [32]. The relationships between various factors and the prevalence of ESBLs in wildlife have been reported in various studies. These factors include high cattle densities [33], the presence of wastewater treatment plants [34], and the existence of scavenger species [35]. Potentially, the bantengs in our study might act as reservoirs hosting ARMs from multiple origins, implying that the ARMs in the population were not solely introduced by the domestic cattle. The results are concerning because this banteng population inhabits an area that serves as an important source of freshwater, nourishing the entire north-east region of Thailand and other South-east Asian countries, including Laos, Cambodia, and Southern Vietnam.

## Conclusion

The presence of ARMs in the wild banteng population poses a threat to public health and wildlife conservation. The results of our study highlight the need for further studies to identify contamination sources and establish surveillance programs for monitoring ARM in wild animal populations.

### Data availability

Supplementary data can be available from the corresponding author on a reasonable request.

## Authors’ Contributions

AK, NR, TPh, TPu, PM, and NS: Performed animal tracking by foot to collect samples. MY, TPh, PM, and NS: Conducted laboratory work. MY, AK, and TPu: Conceptualized the study and data analysis. TPu: Acquired funding and finalized the manuscript. All authors have read, reviewed, and approved the manuscript.
